# Brain autopsies of critically ill COVID-19 patients demonstrate heterogeneous profile of acute vascular injury, inflammation and age-linked chronic brain diseases

**DOI:** 10.1186/s40478-022-01493-7

**Published:** 2022-12-17

**Authors:** Sonal Agrawal, Jose M. Farfel, Konstantinos Arfanakis, Lena Al-Harthi, Tanner Shull, Tara L. Teppen, Arnold M. Evia, Mayur B. Patel, E. Wesley Ely, Sue. E. Leurgans, David A. Bennett, Rupal Mehta, Julie A. Schneider

**Affiliations:** 1 Rush Alzheimer’s Disease Center, Rush University Medical Center, Jelke Building, 1750 W. Harrison Street, Chicago, IL 60612 USA; 2 Department of Pathology, Rush University Medical Center, Chicago, IL USA; 3 Department of Neurological Sciences, Rush University Medical Center, Chicago, IL USA; 4 Department of Biomedical Engineering, Illinois Institute of Technology, Chicago, IL USA; 5 Department of Microbial Pathogens and Immunity, Rush University Medical Center, Chicago, IL USA; 6 Critical Illness, Brain Dysfunction, and Survivorship (CIBS) Center, Vanderbilt University Medical Center, Nashville, TN USA; 7 Center for Health Services Research, Vanderbilt University Medical Center, Nashville, TN USA; 8 Departments of Medicine, Vanderbilt University Medical Center, Nashville, TN USA; 9 The Geriatric Research Education Clinical Center (GRECC), Nashville Veterans Affairs Medical Center, Tennessee Valley Healthcare System (TVHS), Nashville, TN USA

**Keywords:** Autopsy, Severe acute respiratory syndrome coronavirus 2, COVID-19, ICU, Infarct, Inflammation, Lymphocytes, Vasculitis, Acute hemorrhagic leukoencephalopathy

## Abstract

**Background:**

This study examined neuropathological findings of patients who died following hospitalization in an intensive care unit with SARS-CoV-2.

**Methods:**

Data originate from 20 decedents who underwent brain autopsy followed by *ex-vivo* imaging and dissection. Systematic neuropathologic examinations were performed to assess histopathologic changes including cerebrovascular disease and tissue injury, neurodegenerative diseases, and inflammatory response. Cerebrospinal fluid (CSF) and fixed tissues were evaluated for the presence of viral RNA and protein.

**Results:**

The mean age-at-death was 66.2 years (range: 26–97 years) and 14 were male. The patient’s medical history included cardiovascular risk factors or diseases (n = 11, 55%) and dementia (n = 5, 25%). Brain examination revealed a range of acute and chronic pathologies. Acute vascular pathologic changes were common in 16 (80%) subjects and included infarctions (n = 11, 55%) followed by acute hypoxic/ischemic injury (n = 9, 45%) and hemorrhages (n = 7, 35%). These acute pathologic changes were identified in both younger and older groups and those with and without vascular risk factors or diseases. Moderate-to-severe microglial activation were noted in 16 (80%) brains, while moderate-to-severe T lymphocyte accumulation was present in 5 (25%) brains. Encephalitis-like changes included lymphocytic cuffing (n = 6, 30%) and neuronophagia or microglial nodule (most prominent in the brainstem, n = 6, 30%) were also observed. A single brain showed vasculitis-like changes and one other exhibited foci of necrosis with ball-ring hemorrhages reminiscent of acute hemorrhagic leukoencephalopathy changes. Chronic pathologies were identified in only older decedents: 7 brains exhibited neurodegenerative diseases and 8 brains showed vascular disease pathologies. CSF and brain samples did not show evidence of viral RNA or protein.

**Conclusions:**

Acute tissue injuries and microglial activation were the most common abnormalities in COVID-19 brains. Focal evidence of encephalitis-like changes was noted despite the lack of detectable virus. The majority of older subjects showed age-related brain pathologies even in the absence of known neurologic disease. Findings of this study suggest that acute brain injury superimposed on common pre-existing brain disease may put older subjects at higher risk of post-COVID neurologic sequelae.

**Supplementary Information:**

The online version contains supplementary material available at 10.1186/s40478-022-01493-7.

## Introduction

Severe Acute Respiratory Syndrome Coronavirus 2 (SARS-CoV-2) is the third member of the coronavirus family capable of infecting humans and the etiologic agent of coronavirus disease of 2019 (COVID-19). SARS-CoV-2 rapidly spread internationally and resulted in significant morbidity and mortality [1, 2]. Clinical and epidemiological studies have established that a spectrum of neurological manifestations are associated with COVID-19 infection, including seizures, headache, anosmia, and ageusia, acute cerebrovascular accidents, acute necrotizing hemorrhagic encephalopathy, viral meningitis, acute disseminated postinfectious encephalomyelitis, post-infectious brainstem encephalitis, and Guillain–Barre´ and Miller Fisher syndromes [3, 4]. However, there is a debate on how SARS-CoV-2 affects the brain and induces neurological sequelae.

Neuropathological assessment is the gold standard method to elucidate the underlying pathophysiology of disease in the brain. While autopsy studies have reported the neuropathological changes of patients with SARS-CoV-2 infections [5–8], evidence linking SARS-CoV-2 virus directly to the COVID-19 associated neuropathology is debatable. Few studies identified neurotropism and direct neuro-invasive capacity of SARS-CoV-2 to enter the brain [9, 10], while others support the indirect mechanisms mediated by cytokines through systemic effects [11, 12]. Better characterization of both acute and chronic neuropathological features in persons with documented COVID-19 infection may aid treatment and management strategies for persons who suffer neurological manifestations in association with SARS-CoV-2 infection.

Here, we examined autopsy findings from 20 ICU patients with confirmed SARS-CoV-2 acute infection and summarize the clinical, neuroimaging, and histopathological findings that involved the brain.

## Methods

### Participants

The study was approved by the institutional review board of Rush University Medical Center and Vanderbilt University Medical Center. The study was conducted per IRB approved protocol at Vanderbilt University (IRB 192,003). The study used convenience sampling to include consecutive critically ill individuals with confirmed COVID-19 infection whose legally authorized representative (parents/caregiver) were willing to consent to brain tissue donation immediately upon death. Twenty patients were admitted or transferred to the intensive care unit in the middle Tennessee area (e.g., Vanderbilt, Nashville Veterans Affairs Hospital, Summit Regional Hospital, Alive Hospice). After death, brain removal and tissue collection for clinic-neuropathologic research were performed between April 2020-July 2021. Demographics and clinical data were obtained through medical record review when available.

### Autopsy

A systematic autopsy procedure was performed from multiple medical examiner’s office sites in Nashville for cerebrospinal fluid (CSF) collection and brain harvesting as described elsewhere [29]. In brief, a needle was inserted through the corpus callosum into the lateral ventricle of the brain to extract between 8 and 10 mL of fluid, which was frozen at − 80 °C until additional processing. CSF was collected from only 11 COVID-19 subjects. After CSF collection, the brain was harvested, immersion fixed in 4% paraformaldehyde for at least 30 days, and transferred to Rush Alzheimer’s Disease Center, Rush University Medical Center, Chicago where systematic *ex-vivo* postmortem brain MRI and neuropathological evaluations were performed.

### Ex vivo postmortem brain MRI data acquisition

After brain fixation for a minimum of 30 days, ex vivo brain MRI data were acquired using a Siemens 3 Tesla MRI scanner and two sequences: a 2-dimensional multiecho spin echo sequence and a 3-dimensional multiecho gradient echo sequence. Briefly, white matter hyperintensity (WMH) was assessed based on T2-weighted images and WMH burden was rated in periventricular and deep white matter regions according to the original 4-level Fazekas scale. The whole-brain WMH score was the maximum of the periventricular and deep white matter regions ratings [13]. EPVS burden in both brain hemispheres and brainstem of each patient were examined based on T2-weighted ex vivo MRI data, using a semiquantitative 4-level scale (none = 0, mild = 1, moderate = 2, severe = 3). EPVS were defined as hyperintense, tubular, 3-dimensional structures. The 4 levels of the rating scale were defined as follows. None: no EPVS; mild: fewer than 10 EPVS; moderate: > 10 EPVS, but not widespread; severe: widespread EPVS as described earlier [14]. Microbleeds were determined when a relatively round, small, hypointense region was observed on the 3-dimensional multi-echo gradient-echo data. At least half of the hypointensity had to be surrounded by brain parenchyma to qualify [15]. Neuroimaging data were generated by a trained reader who was blinded to clinical and pathologic data.

### Neuropathologic evaluation

After ex vivo postmortem brain MRI, each brain was dissected into hemispheres. Both hemispheres were cut coronally into 1 cm slabs, reviewed for gross pathology, and then blocked for diagnostic purposes. A total of 22 tissue blocks were obtained from multiple brain regions, including midfrontal, middle temporal, entorhinal with amygdala, inferior parietal, calcarine, anterior cingulate, posterior cingulate, anterior temporal tip, inferior orbital frontal cortices, hippocampus, basal ganglia, thalamus, frontal periventricular, parietal periventricular, anterior watershed, posterior watershed, cerebellum, olfactory bulb, substantia nigra, midbrain caudal, pons, and medulla oblongata. Additional blocks with suspected gross pathology, such as infarcts and hemorrhages were also sampled for microscopic examination.

### Histological stains and immunohistochemistry

Paraffin blocks were sectioned at a thickness of 6 µm and slides were stained with hematoxylin and eosin (H&E) to examine histopathological changes including perivascular lymphocytes, acute ischemic changes, microglial nodules, neuronophagia, fibrinoid necrosis, thrombosis, etc. Sections with detected pathology were stained with suitable antibodies when appropriate for validation. The antibodies used in this study are listed in Additional File 1: Table S1.

Immunostaining with HLA-DR antibody was performed on sections of the olfactory bulb and medulla to determine the microglial activation. In addition, immunostaining with anti-CD20, anti-CD4, and anti-CD8 antibodies was performed on medulla and parenchyma from the olfactory region to identify B or T lymphocytes infiltrating from meninges and parenchymal vasculature. A semi-quantitative approach was used to determine the severity of microglial activation and lymphocyte accumulation and graded the severity into none, mild, moderate, or severe [5]. We defined microglial activation severity by enlargement of cell soma and thickening of processes. All tissue samples were examined at the same time by expert raters including at least two board-certified neuropathologists (JAS, RIM) who were blinded to other pathology and demographics.

### Neurodegenerative pathology

Modified Bielschowsky silver stained sections were used to count neuritic plaques, diffuse plaques, and neurofibrillary tangles from the midfrontal, middle temporal, entorhinal, inferior parietal cortices, and hippocampus to classify AD pathology, including the Consortium to Establish a Registry for Alzheimer’s Disease (CERAD) system [16] and Braak staging [17]. Thal phases (Aβ immunohistochemistry) were determined by Aβ immunohistochemistry [18]. National Institute on Aging-Alzheimer’s Association criteria was used to determine the pathologic diagnosis of AD that required either intermediate or high likelihood of AD-neuropathologic change [18]. For assessment of Lewy bodies, α-synuclein immunostains (Zymed; 1:50) from the following brain areas were used: midfrontal, middle temporal, inferior parietal, anterior cingulate, and entorhinal cortices, amygdala, and midbrain. A modified McKeith criteria was used to determine Lewy body dementia pathology (nigra-predominant type/limbic-type/neocortical-type/amygdala-predominant type [19]. Nigral neuronal loss was assessed from 6 µm section of the H&E stained substantia nigra using a semiquantitative scale. The pathologic diagnosis of PD required at least moderate neuronal loss and the presence of Lewy bodies in the nigra [20]. Limbic predominant age-related TDP-43 encephalopathy-neuropathologic changes (LATE-NC) pathology was assessed using immunostaining with a monoclonal antibody to phosphorylated TDP-43 (pS409/410; 1:100) from 8 brain regions: the amygdala, hippocampus, dentate gyrus, and entorhinal, midfrontal, inferior orbital, anterior temporal tip, and middle temporal cortices. LATE-NC stages were classified into 4 stages based on previous studies [21, 22]. Finally, LATE disease was determined using LATE-NC diagnostic criteria that required either LATE-NC stage 2 or stage 3. Hippocampal sclerosis was evaluated from the mid hippocampus and anterior hippocampus bilaterally [22, 23].

### Cerebrovascular pathology

Atherosclerosis was assessed semi-quantitatively by gross evaluation of circle of Willis vessels. Arteriolosclerosis was assessed based on concentric hyalinized thickening of the walls of small arterioles with consequent narrowing of the lumen in basal ganglia. Both vessel pathologies were graded using semi-quantitative scale from 0 (none) to 6 (severe), as previously described [24]. Cerebral amyloid angiopathy (CAA) was assessed by immunostaining of meningeal and parenchymal vessels from four cortical regions (midfrontal, middle temporal, inferior parietal, and calcarine cortices) with Aβ antibodies [25]. In addition, whole brain was examined for infarcts and hemorrhages (subdural, subarachnoid, or parenchymal), suspected infarcts or hemorrhages were blocked and microscopically examined for their presence with definitive age (acute, subacute, and chronic). Additional infarcts and hemorrhages invisible to the eye but identified during the microscopic examination were summarized as microscopic infarcts or hemorrhages [24].

### Detection of SARS-CoV-2

SARS-CoV-2 RNA measurement in CSF: First, RNA from 11 CSF donors were isolated using miRNeasy RNA kit (Qiagen, Germantown, MD). The presence of SARS-CoV-2 viral RNA was assessed with CDC 2019-Novel Coronavirus (2019-nCoV) Real-Time RT-PCR Diagnostic Panel primers and probes (Cat: 2019-nCoVEUA-01; Atlanta, GA, USA) and iTaq one-step RT-PCR mix (Bio-Rad, Vancouver, WA, USA). Briefly, 8 mL of isolated RNA from CSF was included with primer/probe mix and one-step RT-PCR master mix. Primers against SARS-CoV-2 nucleocapsid (N1, N2) and RNase P (internal control) were included for analysis (Additional File 1: Table S2). RNA extraction and RT-PCR were performed in accordance EUA protocol for SARS-CoV-2 diagnostic panel (https://www.cdc.gov/coronavirus/2019-ncov/lab/virus-requests.html). Per diagnostic criteria, any Ct values above 37 were considered not positive for the gene of interest.

SARS-CoV-2 RNA measurement in brain: Briefly, RNA was isolated from postmortem samples from twelve brain regions from individuals who had SARS-CoV2 using the Qiagen Viral RNA Extraction kit per manufacturer’s instructions. The RNA quantity for each sample was measured on a Qubit fluorometer and RNA quality and RNA integrity number (RIN scores) were subsequently determined using the Agilent bioanalyzer. RNA samples were then reverse transcribed, and RT-PCR was performed following the CDC approved protocol employing TaqMan reagents and CDC SARS-CoV2 primer sequences for N1 and N2 SARS-Cov2 viral particles. All primers used are listed in Additional File 1: Table S2.

SARS-Cov-2 protein detection in the brain: Immunohistochemistry using antibodies against viral nucleocapsid (catalog numbers [1:500]; SinoBiological, Eschborn, Germany) was performed on 6 µm sections of medulla oblongata and olfactory sections to detect SARS-CoV-2 in brain tissues. All slides were examined by trained examiners, including at least 2 board-certified neuropathologists.

### Statistical analysis

Neuropathologic characteristics were compared across the group of older people (65 year and above) and group of younger people (below 65 year) as well as compared across the clinically defined groups of people with and without vascular risk factors or people with and without ICU related events by Fisher exact tests. Statistical analyses were programmed in SAS/STAT software, version 9.4 (SAS Institute Inc). A nominal threshold of 2-sided *P* < 0.05 for statistical significance was used.

## Results

### Demographic and clinical presentation

The mean age-at-death of the 20 COVID patients was 66.2 (SD = 17.01; range 26–97) with half of the patients over 70 years of age, 14 (70%) were men, and 4 (20%) were of Hispanic ethnicity. All twenty patients were admitted to the intensive care unit, of which 80% were admitted for respiratory failure. The average length of ICU stay was 12 days (range 1–30 days) and average mechanical ventilation duration was 7 days (range 0–28 days). Most of our participants (n = 17/20; 85%) had a history of one or more chronic age-related risk factors or diseases. Overall, more than two-thirds of the participants had at least two cardiovascular risk factors and more than half of the decedents (n = 11/20; 55%) had a history of cardiovascular disease (Table 1). Interestingly, all but one of those over the age of 70 had cardiac risk or disease. One-fourth of the participants (n = 5) had a history of dementia and all were over the age of 70. Two participants under 50 years of age and one over the age of 70 had no history of vascular risk, clinically diagnosed disease, or other identified medical conditions. Characteristics of detailed demographics, ICU, and clinical findings are listed in Table 1 and Additional File 1: Table S3.

**Table 1:** Demographic and clinical characteristics of decedents who died from COVID-19 (N = 20)

Characteristic	N = 20
Demographic	
Age at death, mean (SD)	67.3 (16.3)
Male, n (%)	14 (70)
Hispanic, n (%)	4 (20)
Race, n (%)	
White	16 (80)
African-American	3 (15)
Asian	1 (5)
Clinical	
Dementia, n (%)	5 (25)
Cardiovascular risk factors, n (%)	15 (75)
Hypertension, n (%)	14 (70)
Diabetes, n (%)	7 (35)
Dyslipidemia, n (%)	10 (50)
Obesity, n (%)	3 (15)
Cardiovascular disease, n (%)	11 (55)
Coronary Artery Disease, n (%)	5 (25)
Cerebrovascular Disease, n (%)	5 (25)
Immunosuppressant medication, n (%)	3 (15)
Hospital related data	
Number of patients in ICU for respiratory failure, n (%)	16 (80)
Number of patients in ICU for shock, n (%)	2 (10)
Number of patients on mechanical ventilators at least once, n (%)	14 (70)
Average time on mechanical ventilators (days), mean (SD)	6.9 (7.2)
^a^Average length of ICU stay (days), mean (SD)	12 (7.85)

^a^Data missing for 3 participants

### Neuroimaging findings

**Fig. 1:**
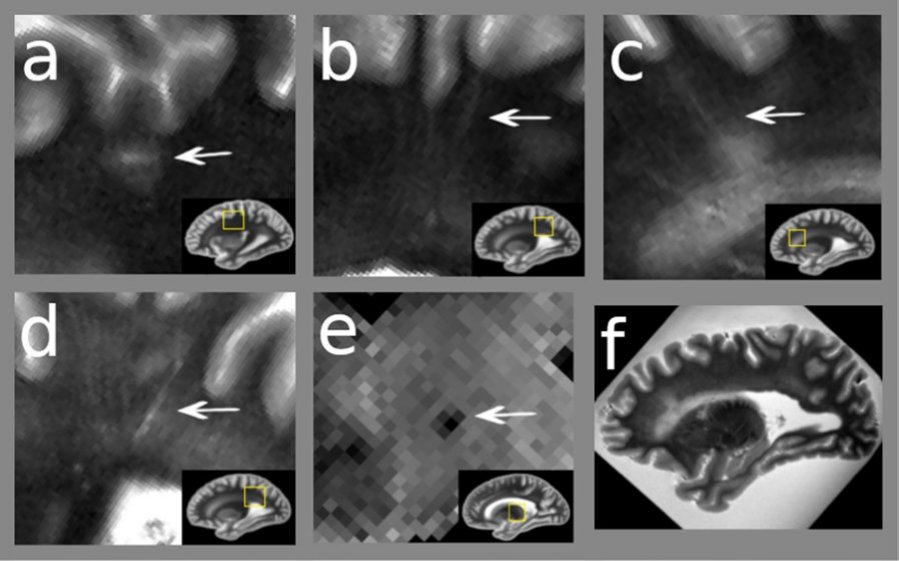
MRI brain findings. **a** MRI image from case 13 shows an acute infarct. **b** EPVS in left hemisphere.** c** and** d**, EPVS in right hemisphere, **e** Microbleed in basal ganglia. **f** White matter hyperintensities in overall brain

About half of the participants (n = 8, 40%) had severe WMH burden, nearly one-fourth (n = 4, 20%) had moderate WMH) burden, and the remaining (n = 7, 35%) had no to mild levels of WMH (Additional File 1: Table S4). One-fourth of the participants (n = 5, 25%) had severe EPVS burden, nearly one-fourth (n = 4, 20%) had moderate EPVS burden, and the remaining (n = 9, 45%) had no to mild levels of EPVS burden (Additional File 1: Table S4). EPVS burden levels in the brainstem were none-to-mild. Older adults (age ≥ 50) and those with a history of cerebrovascular disease or risk factors were more likely to have more moderate-to-severe WMH and EPVS. Moderate-to-severe WMH was more common than any other examined chronic pathology, including AD, PD, Lewy body disease (LBD), limbic predominant age-related TDP-43 encephalopathy neuropathologic changes (LATE-NC), or cerebrovascular disease pathologies. CMB presence was common in the COVID-19 brains; more than 3/4th (n = 16, 80%) of the patients had microbleed (Fig. 1a–f and Additional File 1: Table S4).

### Autopsy findings

Neuropathological examination revealed a range of abnormal acute and chronic pathologies. The median post-mortem interval was 3.98 (SD = 1.34; IQR = 2.66–6.66) hours. Table 2 represents the acute clinicopathologic changes and Table 3 represents the chronic clinicopathologic changes in decedents who died from COVID-19.

**Table 2:** Acute clinicopathologic and neuroinflammatory changes in decedents died from COVID-19 (N = 20)

Case	Age, sex	Cardiovascular disease/risk factors	Recent infarctions and hemorrhages	Acute ischemia	Viral-related changes	Microglial activation	CD4 + T lymphocytes	CD8 + T lymphocytes
1	26M	Yes	Single acute gross SAH	No	No	Mild	MEN - Minimal	MEN - Minimal
							PVS - None	PVS - Moderate
							PAR - None	PAR - Moderate
2	43M	No	No	No	Microglial nodule	Severe	MEN – Minimal	MEN - Minimal
							PVS - Minimal	PVS - Mild
							PAR - None	PAR - Minimal
3	46M	No	Multiple subacute microinfarcts. Multiple acute and subacute gross and micro-IPHs. One acute micro-SAH	Yes	AHLE	Moderate	MEN - Minimal	MEN - Minimal
							PVS - Minimal	PVS - Mild
							PAR - None	PAR - Minimal
4	51M	Yes	Multiple acute gross and microinfarcts	Yes	No	Moderate	MEN - Minimal	MEN - Minimal
							PVS - Minimal	PVS - Minimal
							PAR - None	PAR - Minimal
5	60M	Yes	Multiple acute microinfarcts. Multiple acute gross-IPHs and SAHs	Yes	No	Moderate	MEN - Minimal	MEN - Minimal
							PVS - Minimal	PVS- Mild
							PAR - None	PAR- Mild
6	61F	Yes	No	No	Lymphocytic cuffing	Moderate	MEN - inimal	MEN - Minimal
							PVS - Minimal	PVS - Moderate
							PAR - None	PAR - Moderate
7	63M	Yes	No	No	No	Moderate	MEN - None	MEN - Minimal
							PVS - None	PVS - Mild
							PAR - None	PAR - Mild
8	65F	No	Multiple acute microinfarcts. Multiple acute gross and micro-IPHs and SAHs	No	No	Moderate	MEN - Moderate	MEN - Minimal
							PVS - Minimal	PVS - Minimal
							PAR - Minimal	PAR - Minimal
9	66M	Yes	Multiple acute microinfarcts. Single acute micro-IPH and multiple acute gross-SAHs	Yes	Fibrinoid necrosis	Severe	MEN - Severe	MEN - Minimal
							PVS - Mild	PVS - Minimal
							PAR - Minimal	PAR - Minimal
10	71M	Yes	No	Yes	Lymphocytic cuffing	Severe	MEN - Minimal	MEN - Minimal
							PVS - Minimal	PVS - Mild
							PAR - None	PAR - Mild
11	71M	Yes	Single acute microinfarct	No	No	Severe	MEN - None	MEN - Minimal
							PVS - None	PVS - Mild
							PAR - None	PAR - Mild
12	72M	Yes	Multiple acute gross and micro-IPHs and SAHs	Yes	Lymphocytic cuffing	Severe	MEN - Moderate	MEN - Minimal
							PVS - Minimal	PVS - Moderate
							PAR - Minimal	PAR - Moderate
13	72F	Yes	Single acute microinfarct	No	No	Moderate	MEN - None	MEN - Minimal
							PVS - None	PVS - Mild
							PAR - None	PAR - Minimal
14	77F	Yes	Multiple acute microinfarcts	No	Lymphocytic cuffing	Moderate	MEN - Mild	MEN - Minimal
							PVS - Minimal	PVS - Minimal
							PAR - None	PAR - Minimal
15	77M	Yes	No	No	No	Mild	MEN - Minimal	MEN - Minimal
							PVS - Mild	PVS - Mild
							PAR - Minimal	PAR - Minimal
16	80M	Yes	No	Yes	Neuronophagia	Mild	MEN- Mild	MEN - Mild
							PVS- Minimal	PVS - Mild
							PAR - Mild	PAR - Minimal
17	81M	Yes	Single subacute microinfarct	Yes	Microglial nodule	Mild	MEN - Minimal	MEN - Minimal
							PVS - Minimal	PVS - Mild
							PAR - None	PAR - Mild
18	82M	Yes	Single acute gross IPH	Yes	Microglial nodule	Moderate	MEN - Minimal	MEN - Minimal
							PVS - Minimal	PVS - Mild
							PAR - None	PAR - Mild
19	83F	Yes	Single subacute gross infarct	No	Neuronophagia, lymphocytic cuffing, and vasculitis-like changes	Moderate	MEN - Minimal	MEN - Minimal
							PVS - Minimal	PVS - Mild
							PAR - None	PAR - Minimal
20	96F	No	Single acute microinfarct	No	Microglial nodule and lymphocytic cuffing	Severe	MEN - Minimal	MEN - Minimal
							PVS - Minimal	PVS - Minimal
							PAR - None	PAR - Minimal

*AHLE* Acute hemorrhagic leukoencephalopathy; *IPH* Intraparenchymal hemorrhage; *MEN* Meningeal; *PVS* Perivascular; *PAR* Parenchymal; *SAH* Subarachnoid hemorrhage

**Table 3:** Chronic clinicopathologic findings in decedents died from COVID-19 (N = 20)

Case#	Age (years), Sex	Dementia	Cardiovascular disease/risk factors	Chronic pathologic findings		Neuroimaging findings		
				Neurodegenerative	Vascular	Moderate-to severe WMH	Moderate-to severe EPVS	CMB presence
1	26M	No	Yes	No	No	No	No	Yes
2	43M	No	No	No	No	No	No	No
3	46M	No	No	No	No	No	No	Yes
4	51M	No	Yes	No	No	Yes	No	Yes
5	60M	No	Yes	No	No	No	No	Yes
6	61F	No	Yes	No	No	Yes	Yes	No
7	63M	No	Yes	No	No	Yes	Yes	No
8	65F	No	No	No	No	No	No	Yes
9	66M	No	Yes	DLB limbic-type	Multiple infarcts	Yes	Yes	No
10	71M	Yes	Yes	DLB neocortical-type	No	No	No	No
11	71M	Yes	Yes	PD, DLB neocortical-type, and LATE-NC (stage 3)	No	Yes	Yes	Yes
12	72M	No	Yes	No	ATH, ART, and infarcts	Yes	Yes	Yes
13	72F	No	Yes	No	Multiple infarcts	Yes	Yes	Yes
14	77F	Yes	Yes	No	ART, CAA, and infarct	Yes	Yes	Yes
15	77M	No	Yes	DLB neocortical-type	Multiple infarcts	No	Yes	Yes
16	80M	Yes	Yes	AD and LATE-NC (stage 3)	Single infarct	Yes	Yes	Yes
17	81M	No	Yes	No	No	Yes	No	Yes
18	82M	No	Yes	No	ATH, infarct, and SAH	Yes	Yes	No
19	83F	No	Yes	AD and DLB neocortical-type	Single infarct	Yes	No	Yes
20	96F	Yes	No	AD and LATE-NC (stage 2)	ATH and ART	Yes	No	Yes

*AD* Alzheimer disease; *ART* Arteriolosclerosis; *ATH* Atherosclerosis; *CAA* Cerebral amyloid angiopathy; *CMB* Cerebral microbleed; *DLB* Dementia with Lewy body disease; *EPVS* Enlarged perivascular space; *LATE-NC* Limbic predominant age related TDP-43 encephalopathy neuropathologic change; *PD* Parkinson disease; *SAH* Subarachnoid hemorrhage; *WMH* White matter hyperintensity

### Acute/subacute pathologies

#### Recent ischemic and hemorrhagic lesions

**Fig. 2:**
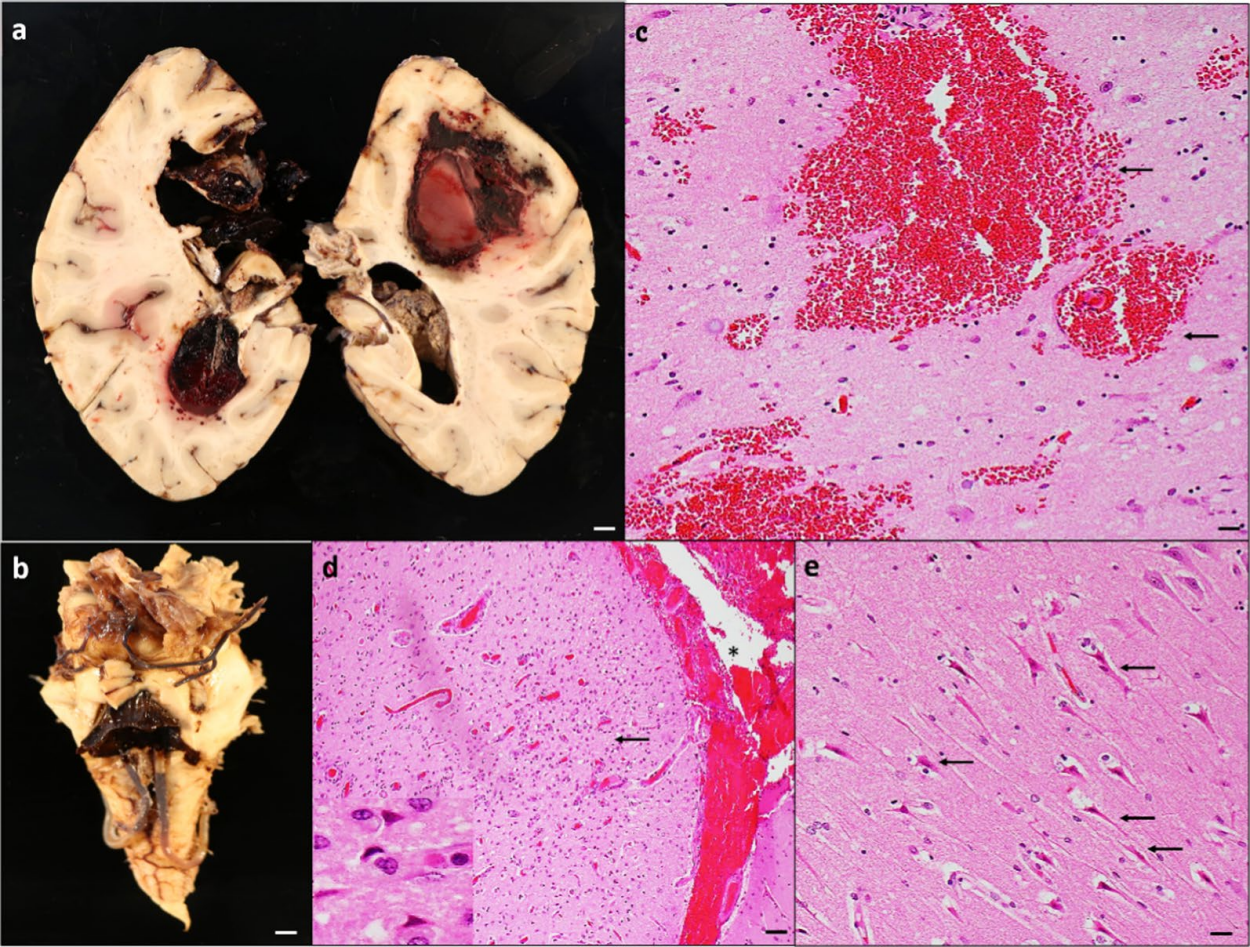
**Acute vascular injuries**. **a** Coronal sections of brain hemispheres show bilateral intraparenchymal hemorrhage in frontal and parietal lobes. **b** The intraparenchymal hemorrhage obliterates the medial left hemisphere and extends into the left lateral, third, and fourth ventricles. **c** Microscopic examination confirms findings in the right parietal region. **d** Image from a H&E-stained section of prefrontal cortex demonstrates acute microscopic infarct (arrow) and acute subarachnoid hemorrhage (*). The inset in **d** shows red neurons, indicative of ischemic change. **e** Section of the hippocampus demonstrates acute ischemic changes (arrows) in CA1 neurons. Scale bar: 1 mm (**a** and **b**), 200 µm (**d**), and 100 µm (**c** and **e**)

Recent ischemic focal lesions were common; 11 brains (55%) showed recent (acute/subacute) ischemic lesions either macroscopic or microscopic. Seven (35%) brains had evidence of macroscopic hemorrhage either in the parenchyma, subarachnoid space, or in both locations (Fig. 2a–d). Of these brains, 3 had both subarachnoid and intraparenchymal hemorrhages, 2 had only intraparenchymal hemorrhages, and 2 had only subarachnoid hemorrhages without evidence of bleeding in the parenchyma. Of those with evidence of macroscopic hemorrhages, 4 (20%) of brains had fresh microscopic hemorrhages either in the parenchyma or in subarachnoid space.

#### Hypoxia/ischemic injury

9/20 (45%) of the brains contained acute hypoxic changes including neuronal shrinkage, red neurons, and pyknotic neurons (Fig. 2e). These findings were predominantly involving the neocortex, CA1 sector of the hippocampus, and Purkinje cells of the cerebellum lateral cortex. Seven out of nine brains with hypoxic-ischemic injury also had either focal recent ischemic, hemorrhagic lesions, or both.

#### Intravascular eosinophilic material

**Fig. 3:**
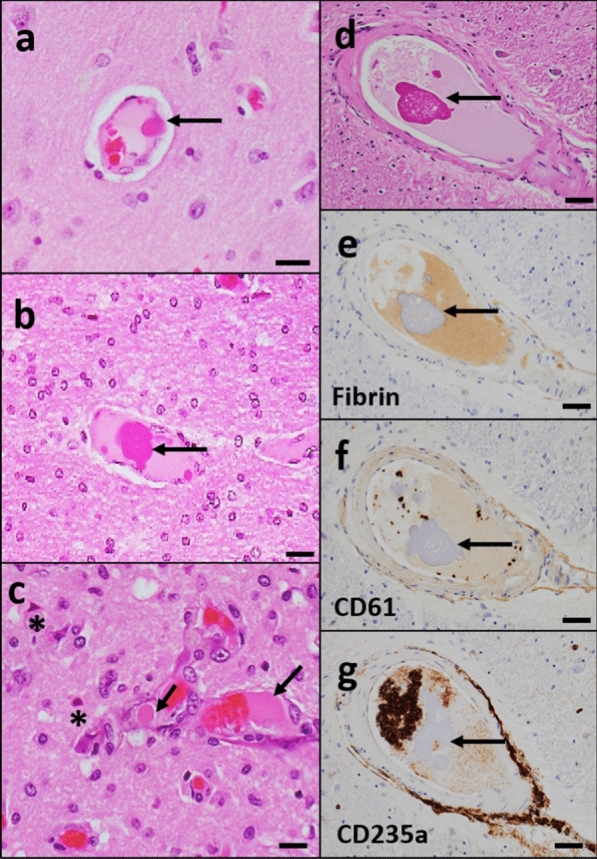
**Amorphous intravascular eosinophilic material**. **a, b** H&E stained section of the mid frontal cortex (**a**) and temporal white matter (**b**) show amorphous eosinophilic globules (arrow) either attached to the vessel wall (**a**) or floated (**b**) in the lumen of the intracerebral blood vessels. **c** H&E-stained section of pre-frontal cortex shows eosinophilic globule (arrow) associated with acute infarct. Red neurons (*) are identified within the infarct area. **d-g** Immunostaining with fibrin (**e**), CD-61 (**f**), and CD235a (**g**) from the thalamus did not stain the eosinophilic globule that was identified in the blood vessel from H&E stained thalamus (**d**). Scale: 100 µm (**d**–**g**) and 50 µm (**a**–**c**)

Intravascular eosinophilic material was observed within arteries or arterioles, either floating in the lumen or attached to the vessel wall in seven brains (35%) (Fig. 3a–d). The material appeared superficially similar to platelet/fibrin aggregates and or microthrombi on H&E-stained sections; however, antigen-specific antibodies (CD61, fibrin, and CD235a stains) were essentially negative, not highlighting these aggregates in vessels arteries, arterioles, and capillaries (Fig. 3e–g). In 3/7 cases, the intraluminal material was associated with acute microscopic infarcts (Fig. 3c).

### Inflammation

#### Microglial nodules and neuronophagia

**Fig. 4:**
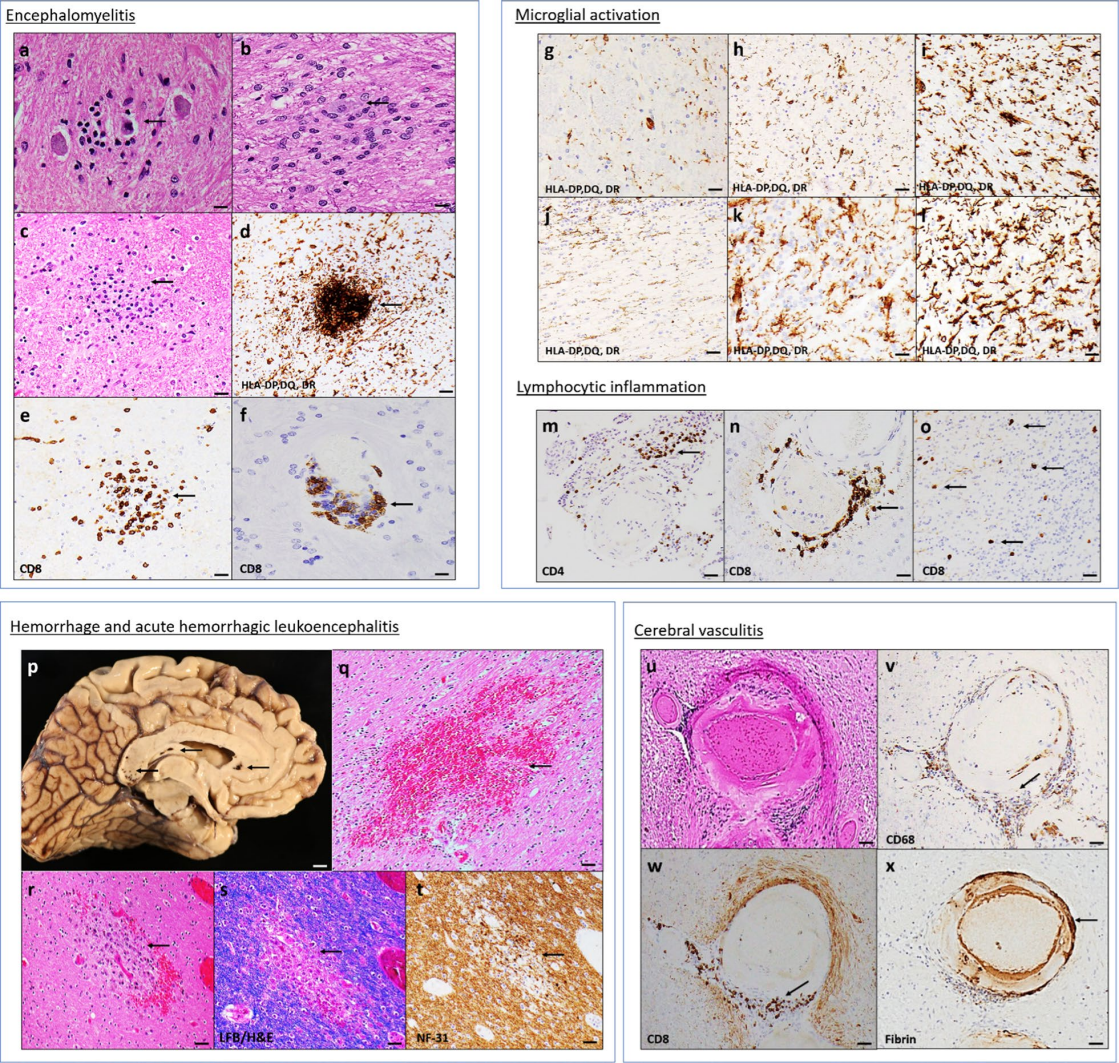
**Inflammatory changes**. Microglial nodules and neuronophagia. **a, b** H&E-stained sections of medulla oblongata at the inferior olivary nucleus **(a)** and cerebellar dentate nucleus **(b)** show neuronophagia (arrow). **c** H&E-stained section of medulla oblongata shows a microglial nodule (arrow). **d** Immunohistochemistry against HLA-DP, DQ, and DR antibody shows clustered microglia in the nodule. **e** Immunohistochemistry against CD8 antibody shows CD8 + T cell accumulation within an area of the microglial nodule. **f** CD8 immunostaining identifies lymphocytic cuffing in the medulla oblongata. Microglial activation. **g-l** Immunostaining with HLA-DP DQ DR shows mild (**g, j**), moderate (**h, k**), and severe (**I, l**) microglial activation from medulla (**g–i**) and olfactory bulb (**j**–**l**). Lymphocytic inflammation. **m** Immunostaining with CD4 shows moderate CD4 + T cell accumulation (arrows) in the meninges. **n, o** Immunostaining with CD8 presents moderate CD8 + T cell accumulation (arrows) around the vessel (**n**) and in the parenchyma (**o**). Acute hemorrhagic leukoencephalitis. p The medial left hemisphere shows multiple petechiae (arrows) throughout the brain including the corpus callosum, primarily in the genu and splenium. **q** H&E stained corpus callosum shows gross acute intraparenchymal hemorrhage. **r** H&E section of the temporal white matter shows necrotic vessel with hemorrhage “ball ring pattern” (arrow) with hemosiderin-laden macrophages, consistent with acute hemorrhagic leukoencephalitis. **s** Luxol-fast blue/H&E stain section demonstrates the perivascular myelin loss (arrow) within the lesion. **t** Immunohistochemistry with neurofilament (NF-31) antibody identifies some damaged axons (arrow) within an area of myelin loss. Cerebral vasculitis-like changes. u H&E section of cerebellar white matter shows vasculitis-related changes (fibrinoid necrosis, disruption of the vessel wall with inflammatory reactions). **v, w** Immunostaining with CD68 and CD8 shows activated microglial cells and macrophages (arrow, v) and T cells (arrow, w) around the vessel wall. **x** Immunostaining with fibrin antibody represents fibrin deposition (arrow) within the vessel wall. Scale bar: 1 mm (**p**), 200 µm (**q**–**x**), 100 µm (**c**–**e** and **g**–**o**), and 50 µm (**a**, **b**, and **f**)

Two (10%) brains showed evidence of neuronophagia (i.e., a dying neuron immediately surrounded by microglial cells) in the locus coeruleus, inferior olivary nucleus of the medulla (Fig. 4a), and cerebellar dentate nucleus (Fig. 4b). H&E stains revealed microglial nodules (i.e., microglia arranged in clusters) in 4 (20%) brains that were confirmed by HLA-DR immunohistochemistry (Fig. 4c–d). A small number of CD8 + T cells were associated with the microglial nodules in all cases (Fig. 4e). The microglial nodules were only noted in the brainstem, where they appeared particularly common in the inferior olivary nucleus and the tegmental nuclei of the medulla and pons and midbrain including the locus coeruleus and midline raphe. Furthermore, H&E stain and CD8 immunohistochemistry revealed lymphocytic cuffings in 6 (30%) brains (Fig. 4f).

#### Microglial activation

Microglial activation was present in the majority of COVID-19 brains. 16 /20 (80%) brains showed moderate-to-severe microglial activation seen either in one region (medulla oblongata/olfactory bulb) or both (Fig. 4g–l).

#### Lymphocytic inflammation

Most of the brains had minimal-mild lymphocytic infiltration around blood vessels, with a few CD8 + or CD4 + T lymphocytes penetrating the meninges and brain parenchyma. Notably, 3 (15%) brains separately showed a significant level of CD4 + T cells in the meninges (Fig. 4m) and 3 (15%) brains showed a moderate amount of perivascular CD8 + T cell infiltrates with a moderate level of CD8 + T lymphocytes penetrating the brain parenchyma (Fig. 4n–o). None of the brains showed B lymphocyte infiltration in either location.

#### Acute hemorrhagic leukoencephalitis (AHLE) in one patient

Lesions consistent with AHLE were found in a 46-year-old patient who was admitted for acute hypoxic respiratory failure in the setting of known COVID-19. Histopathological examination showed typical AHLE-associated pathologic features including infiltration with hemosiderin-laden macrophages and foamy macrophages, astrocytosis, necrosis of small vessels, and hemorrhages in a “ring and ball” pattern (Fig. 4r). Luxol fast blue/H&E (LFB/H&E), a special stain for myelin showed circumscribed perivascular demyelination and neurofilament-31 (NF-31) immunostains further revealed preserved axonal processes within these lesions (Fig. 4s, t). The adjacent tissue was well preserved without necrotic blood vessels and/or perivascular inflammation. These lesions were identified in the white matter location of parietal, temporal, and cerebellar regions. Notably, the brain also showed multiple acute intraparenchymal hemorrhages (Fig. 4p, q) and subacute microinfarcts with microglial activation and CD4 + and CD 8 + T cell accumulation in the brain parenchyma (patient #3, Table2).

#### Vasculitis in one patient

A single brain exhibited vasculitis-like changes histologically defined as fibrinoid necrosis of vessel wall or destruction of vessel wall with inflammatory cells within vessel walls. Histopathological changes were noted in the multiple vessels of the thalamus and cerebellar white matter. H&E stain showed fibrinoid necrosis of vessels wall with inflammation leading vessel wall destruction (Fig. 4u). CD68 and CD8 immunostaining demonstrate the activated macrophages and T lymphocytes, respectively within and around the vessel walls (Fig. 4v-w). Immunostaining with a fibrin antibody confirmed the presence of fibrin in the vessels (Fig. 4x). Of note, the brain of this 83-year-old female also showed subacute infarct, neuronophagia, CD8 + and CD4 + T lymphocytes, and age-related chronic pathology including AD, neocortical Lewy body disease, atherosclerosis, and chronic infarcts (patient #19, Tables 2 and 3).

### Chronic age-related pathologies

Chronic neuropathologies were found in over half of the brain (11 of 20) with 7 brains showing neurodegenerative pathologies and 9 showing vascular pathologies (significant vessel disease and/or infarcts). Lewy body disease was the most common neurodegenerative pathology (n = 5) whereas chronic infarcts were the most common vascular pathology (n = 8). Chronic neurodegenerative or vascular neuropathologies were present in all except one of the 12 cases over the age of 65 years (n = 12), whereas they were absent in those under 65 years. Mixed non-AD neurodegenerative and vascular pathologies were common, present in 5 brains, with mixed limbic/neocortical Lewy body disease and vascular the most common (n = 5/5). Mixed AD and vascular pathology was also common in those over the age of 80 years (3/5).

#### Neurodegenerative diseases

Seven brains showed a range of neurodegenerative diseases [AD in 3 patients, LATE in 3, and Lewy body disorders in 5 (i.e. PD and neocortical LBD in 1), neocortical LBD in another 3, and limbic LBD in 1 decedent] (Additional File 1: Fig. S1a-c). These neurodegenerative changes existed in the older patients age ranging from 67 to 97 years. As literature reported that mixed neurodegenerative pathologies are common in older person brains [26], we found 2 brains with AD had also coexisted with LATE-NC pathology, one with AD had coexisted neocortical LBD, and one with PD had cooccurred neocortical LBD and LATE-NC pathology.

#### Chronic vessel disease and tissue injury

Vessel disease pathology was also present; 4 (15%) brains had moderate-to-severe atherosclerosis, 3 (15%) had arteriolosclerosis and 1 (5%) had cerebral amyloid angiopathy (Additional File 1: Fig. S1d–g). Of note, the single brain with moderate CAA had a low level of AD neuropathologic changes (Thal 3, Braak 1, and CERAD Probable) and did not meet the criteria for pathologic diagnosis of AD. Eight (40%) brains had older macroscopic ischemic lesions. Of these, 4 (20%) had additional microscopic older lesions (Additional File 1: Fig. S1h). None of the brains showed older parenchymal hemorrhage. Only a single brain had older subarachnoid hemorrhage overlying the frontal cortex.

#### qRT-PCR and immunohistochemistry findings for SARS-CoV-2 virus

SARS-CoV-2 RNA and proteins were not detected in any of the 20 patients by qRT-PCR and immunohistochemistry. Viral RNA was also not detected in CSF from eleven patients.

### Clinical-pathologic-imaging observations:

Vascular risk factors or vascular diseases were extraordinarily common in those 65 years of age and above and in those under the age of 65 (n = 11/13, 85% vs n = 5/7, 71%, *P* = 0.586). Chronic vascular brain pathologies were present in over two thirds of those 65 years of age and above (n = 9). In spite of vascular risk factors or disease in more than half of those under the age of 65, none of these younger decedents had chronic vascular brain pathologies showed a common incidence of chronic vascular pathologies in older people than younger people (p value = 0.004). Acute and subacute intraparenchymal infarcts and hemorrhages were common in those with and without vascular risk factors groups (n = 11/16, 69% vs n = 3/4, 75%, *P* value = 1) and those with and without chronic brain pathologies (n = 9/11, 82% vs n = 6/8, 75%, *P* value = 1). Also, acute vascular changes were present in both the young (n = 4/7, 57% in those aged below 65 years) and older age (n = 10/13, 78% in those aged 65 years and above) groups (*P* value = 0.61). Given that recent ischemic lesions can be related to reason for ICU admission, such as respiratory insufficiency, or need for mechanical ventilation, we examined the correlation between these ICU related events with recent ischemic lesions. We found that acute and subacute infarcts and hemorrhages were common in those with and without admission to ICU for respiratory failure (n = 11/16, 69% vs n = 2/4, 50%, *P* value = 0.586) and those with and without mechanical ventilation (n = 9/14, 64% vs n = 4/6, 66%, *P* value = 1).

Of those decedents with dementia (n = 5), all were found to have either neurodegenerative (n = 2), vascular (n = 1), or mixed neurodegenerative vascular pathologies (n = 2) contributing to their dementia syndrome as noted above. Of the older patients without a known history of dementia (n = 8), six had chronic vascular or neurodegenerative neuropathologies. Specifically, 3 had chronic vascular brain pathologies, 3 had mixed vascular and neurodegenerative pathologies and two had no brain pathology. None of the younger subjects had a pathologic diagnosis of Alzheimer’s disease using NIA-AA criteria of intermediate or high ADNC or other dementia-related neurodegenerative or vascular pathologies. Neuronal neurofibrillary tangles in the mesial temporal lobe described as primary age-related tauopathy (PART; Thal = 0 and Braak = 1–4) were seen in 4 decedents (ages of 51, 65, 71, and 81). Only one of these had dementia (age 71) but this decedent also had other neurodegenerative pathologies including LATE, PD, and dementia with Lewy body disease to explain dementia.

Neuroimaging captured additional brain pathology such as WMH, CMB, and EPVS. Because these pathologies could represent recent or chronic brain changes [27, 28], we separately examined their relationship with clinical conditions. EPVS, WMH and CMB pathologies were common in both older and younger groups (EPVS = 7/13; 54%, vs 2/7, 28%, p value = 0.374, WMH = 9/13; 69% vs 3/7, 43%, *P* value = 0.356, CMB = 11/13, 85% vs 4/7; 57%, *P* value = 0.289). Interestingly, EPVS was seen only in those with vascular risk factors (n = 9/16; 56%) while WMH was appeared to be more common in those with vascular risk factors groups compared to those without vascular risk factors groups (n = 11/16; 69% vs n = 1/4; 25%), but their association did not reach to statistical significance (p value = 0.255). CMB was found to be common in both with and without vascular risk factors groups (n = 12/16, 75% vs n = 3/4, 75%, *P* value = 1).

Inflammatory patterns were heterogeneous. Microglial activation and T-cell lymphocytes were not related to age, sex, or other clinical or neuropathological findings. Encephalitis-like changes (microglial nodule, neuronophagia, and lymphocytic cuffings) were appeared to be more common in older age groups than in younger age groups (n = 8/13, 62% vs n = 2/7, 29%, *P* value = 0.349) and in those with chronic brain pathologies than in those without having chronic brain pathologies (n = 7/11, 64% vs n = 3/9, 33%, *P* value = 0.369), but they did not reach to statistical significance (all 2 values > 0.34). The lack of statistical significance could be due to small sample size.

## Discussion

This autopsy series of 20 brains of decedents with acute COVID-19 infection, shows that acute and chronic neuropathologic changes are very common in those with fatal COVID-19 infections. Acute changes are most commonly vascular in origin, with global hypoxia, acute infarcts, and hemorrhages. Inflammation is also common and heterogeneous, with a small but notable number of decedents showing mild or subacute encephalitis-like changes. This autopsy study reports two cases with severe inflammatory reactions, one histopathologically confirms the occurrence of cerebral vasculitis-like changes and another confirms the AHLE. No viral RNA and viral protein in the CSF or brains were detected. Although the tissue was heavily fixed, as a safety precaution, which compromised the quality of brain RNA isolated, the lack of SARS-CoV-2 detection in the CSF of a subset of subjects combined with lack of SARS-CoV-2 protein detection in the brains underscores the findings that the virus was not found in the brain of these subjects. Chronic neuropathologic changes (neurodegenerative, vascular, and mixed pathologies) were also very common, but only in the older decedents where it was common even in the absence of a reported neurologic syndrome.

The results of this study confirm and extend prior COVID-19 autopsy studies in several ways. First, other COVID-19 neuropathologic studies have reported the common occurrence of cerebral hemorrhage, focal spongiosis, and diffuse or focal ischemic necrosis [5, 6, 29, 30]. The current study extends this by showing that these changes are common in those with and without vascular risk factors or disease and in those with and without chronic age-related brain diseases. Interestingly, two brains had recent ischemic pathologies even in the absence of vascular risk factors and underlying chronic brain pathology, suggesting that acute vascular injury may result from direct aspects of SARS-CoV-2 infection or as a result of critical illness in COVID-19. Second, like other COVID-19 neuropathologic studies, inflammatory changes including the presence of microglial nodules, neuronophagia, and microgliosis with some degree of CD4 + and CD8 + T lymphocytes infiltrating in meninges were observed in six decedents [5–7, 31, 32]. This finding is consistent with mild meningoencephalitis in COVID-19 cases. The neuronophagia may indicate test failure for virus detection or, alternatively, autoimmune phenomena. In addition, a few lesions with occasional microglial nodules also contained cytotoxic T lymphocytes in close vicinity to microglia which suggests that microglial cells may activate lymphocytes and potentially induce T-cell stimulation. Third, we found the predominant localization of microglial nodules and neuronophagia in the brain stem region mainly within the medulla oblongata. Many other SARS-CoV-2 studies supported these findings [5, 6, 33] while some have been reported in the hippocampus and frontal cortex [34]. The preferential localization of microglial nodules and neuronophagia in brain stem regions may be associated with the development of breathing difficulties with COIVD-19 as the brainstem is a key structure for various brain functions, including regulation of cardiac and respiratory functions, consciousness, and sleep cycles [35]. Fourth, we did not find CSF or brain viral RNA or protein even though microglial nodules and neuronophagia were present in the COVID-19-infected brains. To date, the presence of SARS-CoV-2 virus in the brain is not consistent across studies, most studies did not confirm presence of virus within the brain [29, 31]while others report very low levels of detectable SARS-CoV-2 RNA and viral protein brains [5, 6]. Fifth, we document the histological evidence of vasculitis-like changes and AHLE in COVID-19 brains. Finally, as expected, age-related neurodegenerative and chronic vascular pathologies were commonly found in COVID-19 older brains, as reported in other studies [5]. Older decedents with a history of dementia, all had either neurodegenerative, vascular, or mixed neurodegenerative vascular pathologies to explain cognitive impairment and dementia. While complete neurological histories were not accessible in all subjects, these pathologies were also present in most of those who did not report dementia in their clinical history. Overall, these clinical-pathologic data support vascular risk factors as a common underlying risk factor and acute vascular pathologies as a common sequelae of severe COVID-19 infection. Whether older subjects with subclinical age-related neurodegenerative and vascular pathologies may also have an increased risk of fatal infection could also be considered. Because of the frequency of acute brain injury, on the background of existing brain disease, these data may have more profound implications for older compared to younger subjects who survive COVID-19 infection.

With regard to acute and subacute ischemic lesions, these may be consistent with thrombotic or thromboembolic events and reperfusion as reported in other studies [5, 6]. In addition, we noticed intravascular microthrombi (eosinophilic amorphous material) of unclear etiology in 7 brains and these were associated with ischemic lesions in 3/7 brains. Immunostaining with antigen clones directed toward platelet, fibrin, and RBC did not highlight these thrombotic aggregates. Few studies depict the presence of platelet microthrombi by CD61 immunostaining in COVID autopsy cases [32, 36]. One of the studies displayed H&E-stained microthrombi that visually look very similar to what was seen in the present study [36]. The lack of photographic evidence of H&E-stained microthrombi in the other study [32] precludes comparison of the eosinophilic material between these studies. Another possibility is that these vascular inclusions could be related to hydrophilic polymer embolism (HPE) which has been demonstrated now in numerous reports and is established as a potentially fatal iatrogenic disease [37]. Further study with a specific focus on these aggregates may help to understand the origin of potential intravascular aggregates seen in COVID-19 brains.

We found vasculitis-like changes included fibrinoid necrosis and inflammation (T lymphocytes, microglia, and macrophages) in multiple vessel walls from one COVID-19-infected brain. This finding is consistent with other neuroimaging studies that reported imaging appearances suggestive of vasculitis in COVID-19 patients, but with lack of pathological confirmation [38–40]. The presence of subacute infarct and white matter changes could be related to complications of cerebral vasculitis in this brain. The presence of vasculitis-like changes suggests either direct endothelial damage caused by a viral attack or indirect inflammatory process [41]. The lack of infectious agent in the brain is probably more suggestive of indirect damage. Finally, this finding provides neuropathological evidence of CNS vasculitis-like changes in the clinical spectrum of SARS-CoV-2 infection and highlights the need for clinicians to be vigilant for vasculitis and its complications in cases of SARS-CoV-2 infection.

This case series also showed a single case with multiple small foci of necrotizing vessels and hemorrhages in a ball and ring pattern with associated myelin loss in the deep white matter of multiple locations, most consistent with a pathological diagnosis of AHLE. AHLE is often considered within the spectrum of acute disseminated encephalomyelitis (ADEM) with higher morbidity and mortality. It has been reported in COVID-19 patients, mostly by neuroimaging studies [42–44] and relatively less often confirmed with neuropathologic autopsy studies [45]. The loss of myelin, the hemorrhage centering around vessels, and the lack of immunohistochemical staining for SARS-CoV-2 protein or RNA, suggests an indirect pathway such as primary vascular or parainfectious or a mechanistic pathway secondary to systemic inflammation and coagulopathy, as compared to direct viral infection [45]. The parainfectious pathway typically occurs after a latent period following a viral illness. The prolonged course of this patient (13 days with tested SARS-coV-2 positive) suggests that AHLE may have developed as a secondary disease during his hospitalization after SARS-CoV-2 virus infection. The treatment of AHLE is relatively unexplored in COVID-19 and requires a better understanding of the parainfectious neuropathology of SARS CoV-2 to aid clinicians in the management and timely treatment of this potential complication of COVID-19.

This study has several strengths. Twenty brains across a spectrum of ages were investigated using uniform detailed neuropathologic methods and ex-vivo imaging. Yet, there are also some limitations. This study included severely ill patients who died from COVID-19 in a single state; therefore, it is not generalizable to less severe cases, recovery cases, or the general COVID-19 population. Further studies should examine the whole spectrum of patients with COVID-19 to better understand the brain changes in relation to COVID-19 status. All patients were in the intensive care, providing a caveat to interpreting neuropathology as a direct result of COVID-19 infection. Examination of neuropathology in ICU patients without COVID-19 could lead to a better interpretation that which neuropathological findings result from COVID-19 infection or which result from the effects of the pre-existing illness requiring ICU, or ICU interventions. Indeed, many of the older patients had significant pre-existing chronic comorbidities, which may have an increased the risk of fatal infection and influence our neuropathologic findings to COVID-19 infection. Further studies with neither COVID-19 nor ICU experience patients prior to death are needed to dissect the effects of co-morbidities on the brain from both the effects of COVID-19, pre-existing illness, and the ICU experience.

In summary, this study documents heterogeneous neuropathology profiles in COVID-19 decedents and may provide clues to susceptibility, risk, and outcomes in those with acute COVID-19 infection. Vascular risk factors across age appears to increase susceptibility to severe COVID-19 infection. In addition, clinically evident or subclinical age-related vascular and degenerative diseases in older persons may further increase risk. Outcomes are likely influenced by acute ischemic lesions and hemorrhages, which is the most common brain finding associated with severe COVID-19 infection. Neuroinflammation is also common but subtle and heterogenous in both severity and type. Evidence of mild encephalitis-like changes in some brains without evidence of SARS-CoV-2 RNA and proteins may suggest a secondary or post-infectious autoimmune type of encephalitis in some persons. Less commonly, we found more severe inflammatory changes, including vasculitis-like changes and AHLE. These acute brain changes may result in significant morbidity in survivors of severe COVID-19 infection. Finally, because many older persons who die following COVID-19 infection have underlying neurodegenerative and vascular pathologies even in the absence of known dementia, COVID-19 may have potential to add or potentiate risk of subsequent cognitive decline or other neurologic sequelae. Continued and expanded investigation is necessary to disentangle the pathogenesis of the vascular and inflammatory changes associated with SARS-CoV-2 virus.

## Supplementary Information

**Additional file 1**.

## Data Availability

Raw data are available by request through the Rush Alzheimer's Disease Center Research Resource Sharing Hub https://www.radc.rush.edu/.
