# Supplementary material for: Brain autopsies of critically ill COVID-19 patients demonstrate heterogeneous profile of acute vascular injury, inflammation and age-linked chronic brain diseases
Source: Acta Neuropathol Commun. 2022 Dec 17;10:186. doi: 10.1186/s40478-022-01493-7 (PMC9758667; doi:10.1186/s40478-022-01493-7)
Supplement: Supplementary file 1 — Additional file 1. [file 40478_2022_1493_MOESM1_ESM.docx]

**Supplementary table 1: Primary antibodies used in the study**

| **Antibody** | **Catalogue number** | **Dilution** | **Manufacturer** |
| --- | --- | --- | --- |
| Anti-beta amyloid, 17-24 monoclonal antibody, clone 4G8 | 800701 | 1:9000 | Bio legend, San Diego, CA, USA |
| Anti-human phosphorylated alpha-Synuclein, (Ser129) monoclonal antibody, Clone pSyn#64 | 015-25191 | 1:20,000 | Wako Chemicals, Richmond, VA, USA |
| Purified anti-TDP43 phospho (Ser409/410) antibody, clone 1D3/TDP-43 | 829901 | 1:10,000 | Bio legend, San Diego, CA, USA |
| Anti-fibrin antibody, clone 59D8 | MABS2155 | 1:100 | Millipore Sigma-Aldrich, St. Louis, MO, USA |
| CD61 monoclonal antibody (2F2) | PA0308 | Predilute | Leica Biosystems Inc, Buffalo Grove IL, USA |
| CD235a monoclonal antibody (JC159) | MA5-12484 | 1:200 | Invitrogen, Waltham, MA, USA |
| Anti- CD20 mouse monoclonal antibody, clone L26 | NCL-L-CD20-L26 | 1:200 | Leica Biosystems Inc, Buffalo Grove IL, USA |
| Anti-CD4 rabbit monoclonal antibody, clone EPR6855 | Ab133616 | 1:500 | Abcam, Waltham, MA, USA |
| Anti- CD8 mouse monoclonal antibody, clone 4B11 | CD8-4B11-L-CE | 1:75 | Leica Biosystems Inc, Buffalo Grove IL, USA |
| Anti- CD68 antibody, clone L514H12 | CD68-L-CE | 1:100 | Leica Biosystems Inc, Buffalo Grove IL, USA |
| Anti- human HLA-DP, DQ, DR, antigen mouse monoclonal antibody, clone CR3-43 | M077501-2-HLA DP DQ DR | 1:100 | Agilent technologies, Santa Clara, CA, USA |
| Anti- Neurofilament H(NF-H) phosphorylated antibody, clone SMI31p | 801602 | 1:5000 | Bio legend, San Diego, CA, USA |
| SARS-CoV/SARS-CoV-2 nucleocapsid antibody, rabbit monoclonal antibody | 40143-R001 | 1:500 | Sino Biological, Wayne, PA, USA |
| SARS-CoV nucleoprotein antibody, rabbit polyclonal antibody | 40143-T62 | 1:500 | Sino Biological, Wayne, PA, USA |

**Supplementary table 2: Primers used in the study-**

| **Primer** | **Sequence (5’ -> 3’)** | **Concentration (nM)** | **Part number** | **Primer/Probe Mix per Tube** | **Manufacturer** |
| --- | --- | --- | --- | --- | --- |
| N1 Forward | GACCCCAAAATCAGCGAAAT | 500 | RV202001 | 22.5 nmol | Centers for Disease Control and Prevention (CDC) (Atlanta, GA, USA)  Catalog Number #  2019-nCoVEUA-01 |
| N1 Reverse | TCTGGTTACTGCCAGTTGAATCTG | 500 | RV202001 | 22.5 nmol |  |
| N1 Probe | FAM-ACCCCGCATTACGTTTGGTGGACC-BHQ1 | 125 | RV202001 | 22.5 nmol |  |
| N2 Forward | TTACAAACATTGGCCGCAAA | 500 | RV202002 | 22.5 nmol |  |
| N2 Reverse | GCGCGACATTCCGAAGAA | 500 | RV202002 | 22.5 nmol |  |
| N2 Probe | FAM-ACAATTTGCCCCCAGCGCTTCAG-BHQ1 | 125 | RV202002 | 22.5 nmol |  |
| RNase-P Forward | AGATTTGGACCTGCGAGCG | 500 | RV202004 | 22.5 nmol |  |
| RNase-P Reverse | GAGCGGCTGTCTCCACAAGT | 500 | RV202004 | 22.5 nmol |  |
| RNase-P Probe | FAM-TTCTGACCTGAAGGCTCTGCGCG- BHQ-1 | 125 | RV202004 | 22.5 nmol |  |
| PC | N/A | N/A | RV202005 | N/A |  |

Abbreviations: FAM, 6-carboxyfluorescein (Biosearch Technologies, Inc., Novato, CA, USA); BHQ-1, Black Hole Quencher-1 (Biosearch Technologies, Inc., Novato, CA, USA).

**Supplementary table 3: Additional clinical characteristics of decedents with COVID-19 (N=20)**

| **Case** | **Age**  **(Years),**  **Sex** | **Race** | **Dementia** | **Psychiatric disease** | **CVD** | **Cardiovascular**  **Risk Factors** | **Immuno-suppressed** | **Nursing Home** | **Initial COVID Symptom** | **Admission to ICU for respiratory failure** | **Length of**  **mechanical**  **ventilation**  **(days)** | **Length**  **of ICU**  **stay**  **(days)** | **Charlson Comorbidity Index** |
| --- | --- | --- | --- | --- | --- | --- | --- | --- | --- | --- | --- | --- | --- |
| 1 | 26M | Black | No | No | CHD | Hypertension, diabetes, dyslipidemia, obesity | N | N | Cough, diarrhea | Yes | 9 | 9 | 5 |
| 2 | 43M | White | No | No | No | No | No | No | Fever, malaise, cough | Yes | 28 | 28 | 0 |
| 3 | 46M | White | No | No | No | No | No | No | Fever, malaise | Yes | 13 | 13 | 0 |
| 4 | 51M | White | No | No | CAD | Hypertension, diabetes | Yes | No | Cough, lethargy | Yes | 7 | 8 | 7 |
| 5 | 60M | White | No | No | No | Hypertension, obesity | No | No | SOB | Yes | 10 | 12 | 2 |
| 6 | 61F | White | No | Schizophrenia | Arrythmia | Hypertension, diabetes | No | Yes | Fever, lethargy | No | 7 | 30 | 3 |
| 7 | 63M | Black | No | No | CVD | Hypertension, diabetes | No | No | Involuntary movements, dysarthria | Yes | 2 | 15 | 7 |
| 8 | 65F | Asian | No | No | No | No | Yes | No | SOB | Yes | 16 | 17 | 3 |
| 9 | 66M | White | No | Bipolar disorder | CHF, CVD | Hypertension, obesity | No | Yes | Fever, SOB | Yes | 7 | 9 | 7 |
| 10 | 71M | White | Yes | Mental retardation | Arrhythmia | No | No | Yes | Agitation | No | 0 | NA | 5 |
| 11 | 71M | Black | Yes | No | No | Hypertension, dyslipidemia | No | No | Lethargy | No | 0 | NA | 4 |
| 12 | 72M | White | No | No | No | Hypertension, dyslipidemia | No | No | SOB | Yes | 12 | 13 | 6 |
| 13 | 72F | White | No | No | CVD | Hypertension, diabetes, dyslipidemia | Yes | No | Fever | Yes | 4 | 5 | 8 |
| 14 | 77F | White | Yes | Depression | AA, CAD | Hypertension, dyslipidemia | No | Yes | Fever, altered mentation | Yes | 0 | 5 | 6 |
| 15 | 77M | White | No | No | CAD, CVD | Hypertension, dyslipidemia | No | No | Fatigue, diarrhea | Yes | 4 | 13 | 9 |
| 16 | 80M | White | Yes | Depression | No | Dyslipidemia | No | Yes | Falls | Yes | 0 | 1 | 8 |
| 17 | 81M | White | No | No | No | Hypertension, diabetes,  dyslipidemia | No | Yes | Fever, lethargy | Yes | 14 | 16 | 5 |
| 18 | 82M | White | No | No | CAD | Hypertension, dyslipidemia | No | No | Fever, Fall | Yes | 5 | 6 | 5 |
| 19 | 83F | White | No | No | CAD | Hypertension | No | Yes | Abdominal pain | No | 0 | 4 | 10 |
| 20 | 96F | White | Yes | No | No | No | No | Yes | SOB | Yes | 0 | NA | 5 |

Abbreviations: AA, aortic aneurysm; CAD, coronary artery disease; CHF, congestive heart failure; CHD, congestive heart disease; CVD, cerebrovascular disease; NA, not available; SOB, shortness of breath

**Supplementary table 4: Neuroimaging characteristics of decedents with COVID-19 (N=20)**

| **Case#** | **Age**  **(Years),**  **Sex** | **Race** | **WMH (PV)** | **WMH (DWM)** | **CMB** | **EPVS** |
| --- | --- | --- | --- | --- | --- | --- |
| 1 | 26M | Black | L - None  R - None | L - Mild  R - Mild | L - 0  R- 2 | L - Mild  R – Mild  BC - Mild |
| 2 | 43M | White | L - Mild  R - Mild | L - Mild  R - Mild | L - 0  R- 0 | L - Mild  R - Mild  BC - None |
| 3 | 46M | White | L - None  R - None | L - Mild  R - Mild | L - 3  R- 4 | L - Mild  R - Mild  BC - None |
| 4 | 51M | White | L - Severe  R - Severe | L - Mild  R - Moderate | L - 4  R- 0 | L - Mild  R - Mild  BC- None |
| 5 | 60M | White | Not assessed due to large infarct | Not assessed due to large infarct | L - 3  R - 1 | L - Mild  R - Mild  BC- None |
| 6 | 61F | White | L - Severe  R - Severe | L - Mild  R - Mild | L - 0  R- 1 | L - Moderate  R - Moderate  BC – Mild |
| 7 | 63M | Black | L - Moderate  R - Mild | L - Severe  R - Severe | L - 0  R- 0 | L - Severe  R - Severe  BC- Mild |
| 8 | 65F | Asian | L - Mild  R - Mild | L - Mild  R - None | L - 3  R- 5 | L - Mild  R - Mild  BC- Mild |
| 9 | 66M | White | L - Severe  R – Severe | L - Mild  R – Mild | L - 6  R- 2 | L - Mild  R - Moderate  BC- None |
| 10 | 71M | White | L - None  R – None | L - None  R - None | L - 0  R- 0 | L - None  R - None  BC – None |
| 11 | 71M | Black | L - Mild  R – Mild | L - Mild  R - Moderate | L - 1  R- 1 | L - Severe  R – Severe  BC – None |
| 12 | 72M | White | L - Mild  R – Mild | L - Moderate  R - Moderate | L - 1  R- 0 | L - Severe  R - Severe  BC- None |
| 13 | 72F | White | L - Severe  R - Moderate | L - Moderate  R - Severe | L - 1  R- 3 | L - Severe  R - Severe  BC – Mild |
| 14 | 77F | White | L - Severe  R - Moderate | L - Mild  R – Mild | L - 1  R- 0 | L - Moderate  R – Moderate  BC – Mild |
| 15 | 77M | White | L - None  R – None | L - Mild  R – Mild | L - 1  R- 0 | L - mild  R - moderate BC- mild |
| 16 | 80M | White | L - Severe  R – Severe | L - Severe  R - Moderate | L - 1  R- 0 | L - Severe  R – Severe  BC- Mild |
| 17 | 81M | White | L - Moderate  R - Moderate | L - None  R - None | L - 1  R- 1 | L - Mild  R – Mild  BC- Mild |
| 18 | 82M | White | L - Mild  R – Mild | L - None  R - None | L - 1  R - 0 | L - Mild  R - Mild  BC – Mild |
| 19 | 83F | White | L - Severe  R – Severe | L - Severe  R - Severe | L - 4  R- 3 | L - Mild  R - Mild  BC – Mild |
| 20 | 96F | White | L - Moderate  R - Moderate | L - Mild  R - None | L - 1  R- 0 | L - Mild  R – Mild  BC -None |

Abbreviations: L; left hemisphere, R; right hemisphere, BC; brainstem and cerebellum; WMH; white matter hyperintensity, DWM; deep white matter; PV; periventricular, CMB; cerebral microbleed, EPVS; enlarged perivascular space


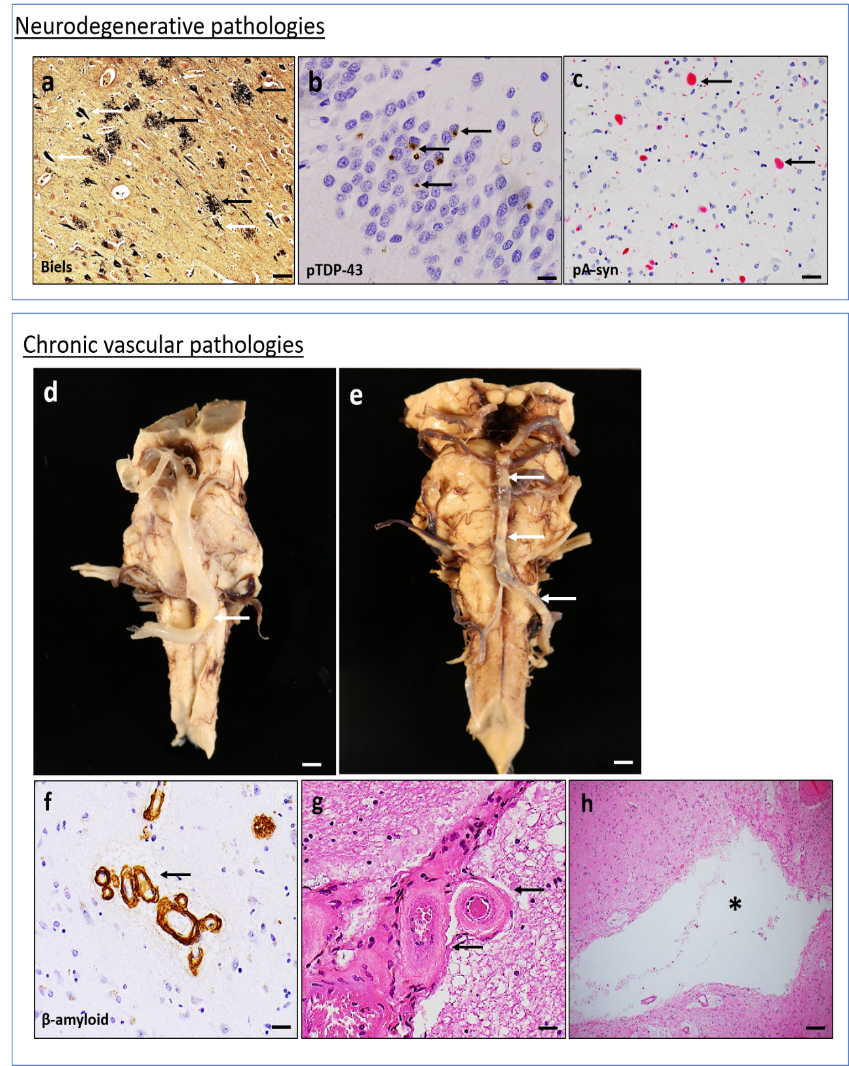
**Supplementary Fig. 1: Age-linked chronic neuropathologies. Neurodegenerative pathologies. a** Modified Bielschowsky stained-section of hippocampus CA-1 sector shows AD-neuropathologic changes including tangles (white arrows) and amyloid plaque (black arrows). **b** Immunostaining with pTDP-43 shows LATE-NC changes like p-TDP-43 cytoplasmic inclusions (arrows) in the dentate gyrus. **c** Immunostaining with pA-syn supports the finding of Lewy body disease (neocortical type by demonstrating Lewy body (arrows) and Lewy neurites in the middle temporal cortex. **Chronic vascular pathologies.** **d, e** Gross examination of Circle of Willis shows minimal atherosclerosis (arrow, d) and moderate atherosclerosis **(arrow, e)**. **f** Immunostaining with 4G8 shows CAA (arrow) in the parenchyma. **g, h** H&E stained section shows moderate-to-severe arteriolosclerosis **(arrows, g)** and chronic (old) microinfarct **(h)** with gliosis and cavitation (*) in the putamen. Scale bars: 1mm (d-e), 200µm (h) and 100µm (a-c, f, and g).
